# Supplementary material for: The Evolution and Characterization of the RNA Interference Pathways in Lophotrochozoa
Source: Genome Biol Evol. 2024 May 7;16(5):evae098. doi: 10.1093/gbe/evae098 (PMC11114477; doi:10.1093/gbe/evae098)
Supplement: evae098_Supplementary_Data [file evae098_supplementary_data.zip › Supplementary_materials.zip!/selected_miRNAs/NoGe_selected_miRNAs/novel_miRNA_families_pdf_files/NMRB01000064.1_Notospermus_geniculatus_strain_Ushimado_scaffold64,_whole_genome_shotgun_sequence_2361.pdf]

Provisional ID	:	NMRB01000064.1_Notospermus_geniculatus_strain_Ushimado_scaffold64,_whole_genome_shotgun_sequence_2361
Score total	:	48686.2
Score for star read(s)	:	3.9
Score for read counts	:	48681.3
Score for mfe	:	1.6
Score for randfold	:	0
Score for cons. seed	:	-0.6
Total read count	:	95498
Mature read count	:	75055
Loop read count	:	0
Star read count	:	20443

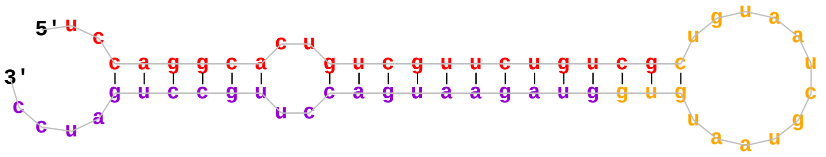

Genome coordinates: NMRB01000064.1 282532-282592

pre-miRNA sequence:
TCCAGGCACTGTCGTTCTGTCGTCCAGGCACTGTCGTTCTGTCGCTGTAATCGTAATGTGGTAGAATGACCTTGCCTGATCC

Mature sequence: TCCAGGCACTGTCGTTCTGTCG

BLAST results for the pre-miRNA sequence against the *Lineus longissimus* genome assembly:

**Scaffold**	**Start**	**Stop**	**Identity**	**e-value**
OU342994.1 1	2625833	2625778	91.071	1.05e-13

```
Nge_novel_miRNA     TCCAGGCACTGTCGTTCTGTCGCTGTAATCGTAATGTGGTAGAATGACCTTGCCTG
Llo_genome          TCCAGGCACTGTCGTTCTGTCGCTGCAATTGAAATGTGGTAGAATGATGTTGCCTG
```
